# Technical and regulatory challenges in cell therapy products: lessons from unsuccessful IND and BLA/NDA approvals

**DOI:** 10.1038/s41420-026-03258-w

**Published:** 2026-07-15

**Authors:** Dongmei Han, Jinping Xie, Yufang Shi, Rong Shao

**Affiliations:** 1 Institute of Regulatory Science for Medical Products, China Pharmaceutical University, Nanjing, China; 2 Department of Biotherapy, The Third Affiliated Hospital of Soochow University, Changzhou, Jiangsu China; 3 Wuxi Sinotide New Drug Discovery Institutes, Huishan Economic and Technological Development Zone, Wuxi, Jiangsu China; 4 Institutes for Translational Medicine, Soochow University, Suzhou, Jiangsu China

**Keywords:** Cell therapies, Cell therapies, Drug regulation

## Abstract

Cell therapy products have advanced rapidly owing to their unique biological advantages and transformative therapeutic potential. However, the inherent complexity of living cell-based products, coupled with disease-specific uncertainties, poses significant technical and regulatory challenges throughout the stringent review and approval process as biologics. This commentary offers an in-depth analysis of current regulatory practices and relevant literature, systematically examining unsuccessful approval scenarios for cell therapy products at both the Investigational New Drug (IND) application stage and the Biologics License Application/New Drug Application (BLA/NDA) stage. Particular attention is given to recurring issues such as manufacturing and quality deficiencies, comparability challenges, and gaps in supporting evidence that frequently lead to clinical holds, delays, or Complete Response Letters. By highlighting these critical failure modes, the article aims to provide actionable regulatory compliance guidance and practical technical risk-mitigation strategies to assist research and development organizations in improving product quality, strengthening regulatory submissions, and enhancing the likelihood of successful development and market authorization.

**Figure:**
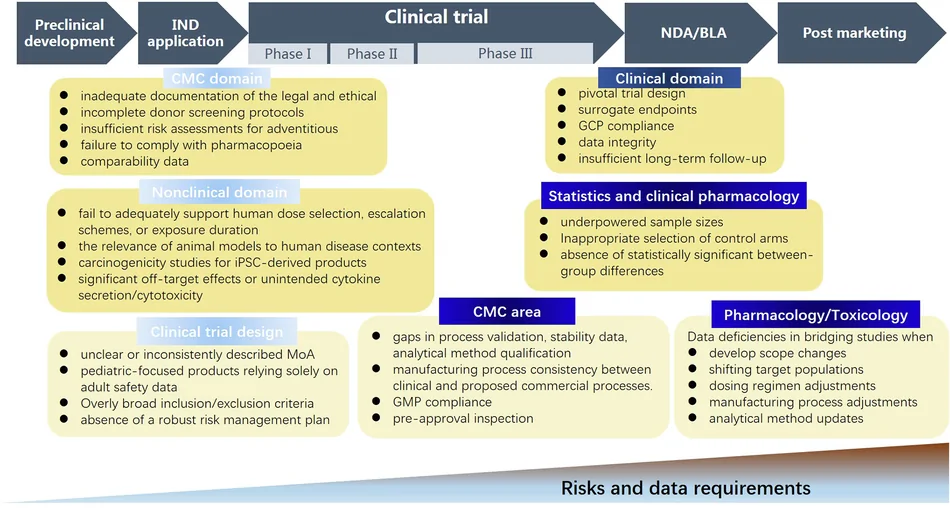
Schematic overview of the key regulatory milestones and common technical/regulatory pitfalls in the development and approval of cell and gene therapy products. The figure illustrates the main stages from preclinical development through IND application, clinical trials (Phases I–III), NDA/BLA submission, and post-marketing surveillance. Major risk areas are highlighted, including CMC, Nonclinical, Clinical trial design, Clinical, Statistics and clinical pharmacology, and Pharmacology/Toxicology domains. For each domain, representative challenges and data deficiencies frequently associated with regulatory delays, clinical holds, or non-approval are summarized. This schematic serves as a practical high-level guide for sponsors navigating the IND and BLA/NDA processes.

Schematic overview of the key regulatory milestones and common technical/regulatory pitfalls in the development and approval of cell and gene therapy products. The figure illustrates the main stages from preclinical development through IND application, clinical trials (Phases I–III), NDA/BLA submission, and post-marketing surveillance. Major risk areas are highlighted, including CMC, Nonclinical, Clinical trial design, Clinical, Statistics and clinical pharmacology, and Pharmacology/Toxicology domains. For each domain, representative challenges and data deficiencies frequently associated with regulatory delays, clinical holds, or non-approval are summarized. This schematic serves as a practical high-level guide for sponsors navigating the IND and BLA/NDA processes.

## Introduction

In the mid-to-late 20th century, breakthroughs in biosciences and genetic engineering ushered in a new era of modern medicine, facilitating the development of both cell-based therapeutics and conventional small-molecule drugs. Cell therapy products stand out due to their superior targeting specificity, favorable drug resistance profiles, flexible dosing regimens, reduced infusion frequency, and the potential for patient-specific customization. These distinctive attributes position them as promising solutions for complex diseases, including malignancies, autoimmune disorders, and degenerative conditions.

As of recent global pipeline analyses, the number of cell therapy candidates under development has expanded substantially, with China accounting for approximately 30% of the worldwide total, ranking second globally (https://healthcareasiamagazine.com/healthcare/news/china-commands-30-global-biopharma-pipeline-drives-asias-share-43). In China, the rapid expansion of preclinical and clinical studies reflects robust governmental support and growing industry investment. However, the complex biological nature of living cells—characterized by variability in source materials, proliferation potential, differentiation capacity, and functional attributes—combined with uncertainties in disease pathophysiology, presents substantial risk management challenges for developers, regulators, healthcare providers, and ethics committees.

Regulatory frameworks in major jurisdictions, including China’s National Medical Products Administration (NMPA) Center for Drug Evaluation (CDE) and the U.S. Food and Drug Administration (FDA), emphasize rigorous evaluation of safety, efficacy, and quality. Drawing on these practices, along with recent literature and real-world case studies, this comment systematically reviews unsuccessful approval scenarios at the IND stage (the gateway to human trials) and the BLA/NDA stage (marketing authorization). The analysis highlights recurring technical and regulatory pitfalls, particularly in Chemistry, Manufacturing, and Controls (CMC), nonclinical toxicology, clinical trial design, and data integrity. Its primary goal is to deliver practical compliance guidance and risk mitigation strategies, enabling research and development organizations to strengthen the rigor of their regulatory submissions and accelerate pathways to successful approval.

Recent FDA data underscore the urgency: between 2020 and 2024, approximately 74% of Complete Response Letters (CRLs) for cell and gene therapies stemmed from quality or manufacturing deficiencies rather than efficacy or safety failures (https://www.drugdiscoverynews.com/why-gene-and-cell-therapies-are-stalling-at-the-fda-16527). A similar pattern is evident in CDE reviews, where CMC inconsistencies and inadequate risk assessments frequently trigger clinical holds or application rejections [1]. As the field matures and regulators refine their expectations for evidentiary standards, understanding these critical lessons is essential for advancing cell therapy development.

## Investigational New Drug (IND) application

The IND application represents the initial regulatory gateway for initiating registered clinical trials. Sponsors must submit comprehensive CMC, nonclinical, and early-phase clinical data to demonstrate that the product is reasonably safe for first-in-human (FIH) administration. Deficiencies in any of these domains commonly result in clinical holds or outright rejection, delaying development timelines significantly.

### CMC domain

In the CMC domain, based on the multiple technical guidelines for cell and gene therapy products issued by the NMPA, which describe the technical requirements for the IND stage, the authors believe that recurring deficiencies include inadequate documentation of the legal and ethical sourcing of starting raw materials, such as donor-derived cells. Issues often arise from incomplete donor screening protocols, insufficient risk assessments for adventitious agents (e.g., viruses, bacteria, or prions), and failure to comply with pharmacopoeia-specified culture methods for sterility and mycoplasma release testing.

Meanwhile, the FDA has introduced some flexibilities in early-stage CMC requirements to accelerate innovation, provided that sponsors maintain strong comparability data (https://www.fda.gov/news-events/press-announcements/fda-increases-flexibility-requirements-cell-and-gene-therapies-advance-innovation).

### Nonclinical domain

In the nonclinical domain, common pitfalls involve pharmacology and toxicology studies that fail to adequately support human dose selection, escalation schemes, or exposure duration. Regulators are increasingly scrutinizing the relevance of animal models to human disease contexts, particularly when bridging nonclinical data to proposed clinical populations. Sponsors are advised to conduct early consultations to ensure study designs address potential species-specific differences and provide clear dose-response relationships.

For induced pluripotent stem cell (iPSC)-derived products, completing carcinogenicity studies prior to first-in-human trials is a mandatory prerequisite for regulatory approval [2]. Toxicology findings may also reveal significant off-target effects or unintended cytokine secretion/cytotoxicity, raising concerns about post-infusion safety and reduced therapeutic efficacy [3].

### Clinical trial design

Regarding clinical trial design, deficiencies frequently stem from an unclear or inconsistently described mechanism of action (MOA), weakening the scientific rationale for development. In FIH trials, imprecise disease diagnostic criteria can hinder accurate patient selection. For pediatric-focused products, relying solely on adult safety data without appropriate bridging strategies or direct pediatric enrollment in early phases raises ethical and scientific concerns.

Overly broad inclusion/exclusion criteria may obscure safety signals, while the absence of a robust risk management plan (including monitoring for cytokine release syndrome (CRS) and immune effector cell-associated neurotoxicity syndrome (ICANS), plus availability of emergency interventions like tocilizumab) can prompt clinical holds.

To mitigate these risks, developers should implement phased risk-based approaches: conduct thorough gap analyses against relevant CDE and FDA guidelines early, engage in pre-IND meetings, and prioritize robust CMC characterization and nonclinical bridging studies. Proactive planning for long-term follow-up and adverse event management is equally essential.

## Biologics license application/new drug application (BLA/NDA)

The BLA/NDA stage marks a critical milestone for marketing authorization. Regulators apply heightened scrutiny, focusing on robust evidence of clinical benefit from pivotal trials and comprehensive long-term safety data. Unsuccessful outcomes most often arise from shortcomings in trial design, execution, statistical robustness, or manufacturing consistency.

### Clinical domain

In the clinical domain, recurring deficiencies include inadequate pivotal trial design, such as failures in randomization, blinding, or use of concurrent controls, resulting in unreliable efficacy and safety evidence (https://investors.atarabio.com/news-events/press-releases/detail/380/atara-biotherapeutics-provides-regulatory-and-business). Notably, substantial questions and uncertainties remain surrounding the regulatory acceptability of surrogate endpoints. For example, initial CAR-T approvals often relied on the objective response rate (ORR). As CAR-T therapies continue to advance from later-line to earlier frontline treatment settings, the FDA has increasingly pivoted toward randomized controlled trials (RCTs) employing time-to-event endpoints, including event-free survival (EFS) or progression-free survival (PFS), as the preferred basis for full marketing approval [4]. Correspondingly, the CDE encourages quantitative validation of surrogate-clinical endpoint relationships to bolster marketing applications [5, 6].

Other issues encompass poor Good Clinical Practice (GCP) compliance, data integrity problems, and insufficient long-term follow-up, particularly for delayed adverse events such as secondary malignancies. Recent examples illustrate these challenges. Atara Biotherapeutics’ tabelecleucel (tab-cel/Ebvallo) for EBV+ posttransplant lymphoproliferative disease faced multiple CRLs, with issues evolving from manufacturing inspections in early 2025 to questions about the adequacy of single-arm trial design and analysis in a subsequent 2026 CRL, despite resolved GMP concerns (https://www.oncologynewscentral.com/drugs/info/fda-delivers-second-rejection-to-car-t-for-posttransplant-lymphoproliferative-disorder).

### Statistics and clinical pharmacology

In statistics and clinical pharmacology, common deficiencies include underpowered sample sizes, absence of statistically significant between-group differences, and flawed benefit-risk evaluations. Inappropriate selection of control arms or failure to demonstrate therapeutic superiority can doom applications. A notable case is BrainStorm Cell Therapeutics’ NurOwn^®^ (debamestrocel), an autologous mesenchymal stem cell therapy for amyotrophic lateral sclerosis (ALS). Following failure to meet prespecified primary and secondary endpoints in the Phase 3 trial, post-hoc subgroup analyses restricted to patients with milder baseline disease were deemed inadequate by the FDA, contributing to the rejection of BLA (https://www.hoganlovells.com/en/publications/unapproved-stem-cell-therapies-remains-a-top-fda-enforcement-priority). The agency underscored the criticality of pre-specified statistical analyses and raised persistent concerns regarding the clarity of mechanism and robustness of manufacturing controls.

### CMC area

According to Technology Networks, in 2025, multiple high-profile cell and gene therapy programs received FDA CRLs primarily due to CMC shortfalls, including gaps in process validation, stability data, analytical method qualification, and facility GMP compliance (https://www.drugdiscoverynews.com/why-gene-and-cell-therapies-are-stalling-at-the-fda-16527). These cases illustrate that even strong clinical efficacy signals can be undermined by unresolved manufacturing deficiencies, emphasizing the need for early alignment with regulatory expectations on comparability and scalability. Similarly, guidelines issued by NMPA CDE, such as the Technical Guideline for CMC Research and Evaluation of Human Stem Cell Products, emphasize parallel requirements for process consistency and rigorous quality control.

CMC issues at this stage frequently center on manufacturing inconsistencies between clinical and proposed commercial processes. Without adequate bridging studies, sponsors cannot demonstrate that commercial product performance matches trial results. Facility GMP compliance shortfalls and unresolved observations from pre-approval inspection (PAI) have stalled multiple programs in 2025, including Capricor Therapeutics’Deramiocel (CAP-1002) for Duchenne muscular dystrophy cardiomyopathy, which received a CRL citing incomplete clinical, CMC, and nonclinical data.

### Pharmacology/Toxicology

In pharmacology/toxicology, deficiencies arise when development scope changes (e.g., adding pediatric populations without conducting juvenile animal studies in accordance with ICH S11 guidelines) or when shifting target populations (e.g., from advanced to earlier-stage disease) renders prior nonclinical data inadequate.

Broader regulatory trends reflect a growing demand for greater rigor in evidentiary standards. However, given that clinical development—particularly for cell and gene therapies—frequently encounters numerous big changes during the trial phase (e.g., manufacturing process adjustments, analytical method updates, or dosing regimen adjustments), the bridgability of nonclinical pharmacology and toxicology studies may become critically important.

## Conclusion

The regulatory landscape for cell therapy products remains dynamic and stringent, reflecting continuous refinement as scientific understanding evolves. Developers face a dual imperative: delivering compelling scientific evidence while navigating the complex biological and manufacturing realities inherent to these therapies. Success hinges on establishing rigorous standards from the IND stage—through robust CMC characterization, ethically sourced materials, comprehensive nonclinical risk assessments, and well-designed early-phase clinical protocols paired with appropriate risk management strategies. At the BLA/NDA stage, the emphasis must shift to pivotal trial integrity, statistical robustness, long-term safety profiles, and manufacturing consistency, all supported by well-executed bridging studies. Key mitigation strategies include early and frequent regulatory engagement (e.g., pre-IND and Type B/C meetings), proactive comparability planning, rigorous validation of analytical methods, and quantitative validation of endpoints when surrogate markers are employed. Lessons gleaned from unsuccessful cases—such as CMC-driven CRLs (e.g., Capricor Deramiocel and Atara tabelecleucel), inadequate trial controls, and overreliance on post-hoc analyses (e.g., NurOwn)—underscore the critical value of pre-specified, rigorous designs and holistic risk-based approaches. By internalizing these insights, research and development organizations can enhance product quality, strengthen the rigor of their regulatory submissions, and increase the probability of timely approvals. Ultimately, addressing these technical and regulatory challenges will help realize the full therapeutic promise of cell therapies for patients worldwide.
